# The psychology of the wait time experience – what clinics can do to manage the waiting experience for patients: a longitudinal, qualitative study

**DOI:** 10.1186/s12913-019-4301-0

**Published:** 2019-07-08

**Authors:** Holly Chu, Robert A. Westbrook, Sarah Njue-Marendes, Thomas P. Giordano, Bich N. Dang

**Affiliations:** 1 School of Allied Health, Baylor College of Medicine, Houston, TX USA; 2 Jesse H. Jones Graduate School of Business, Rice University, Houston, TX USA; 3 VA Center for Innovations in Quality, Effectiveness and Safety (IQuESt), Houston, TX USA; 4 Michael E. DeBakey Veterans Affairs Medical Center, (152); 2002 Holcombe Blvd, Houston, TX 77030 USA; 5 Department of Medicine, Baylor College of Medicine, Houston, TX USA

**Keywords:** Wait time, Patient experience, Patient satisfaction, Patient-centered care, Patient preference, Physician-patient relations, Qualitative studies, Longitudinal studies

## Abstract

**Background:**

Wait time, defined as time spent in the waiting and exam rooms waiting to see a provider, is a key quality metric in a number of national patient experience surveys. However, the literature on wait time does not show a consistent correlation between long waits and worse overall patient care experiences. Herein, we examine contextual factors that can shape the manner in which patients may respond to different wait times. We also identify actions providers and clinics can take to promote positive wait experiences and mitigate negative ones.

**Methods:**

We conducted over 130 h of semi-structured interviews with patients new to two HIV primary care clinics in Houston, Texas. We interviewed patients before the first provider visit, again within two weeks of the first visit, and again at 6–12 months. We analyzed the interviews using directed and conventional content analysis.

**Results:**

Our study showed that patients’ “willingness to wait” is the product of the actual wait time, individual factors, such as the perceived value of the visit and cost of a long wait, and clinic and provider factors. Analyses revealed key steps providers and clinics can take to improve the wait time experience. These include: 1) proactively informing patients of delays, 2) explicitly apologizing for delays, and 3) providing opportunities for diversion. Patients noted the importance of these steps in curtailing frustrations that may result from a long wait.

**Conclusions:**

Our study highlights key steps cited by patients as having the potential to improve the wait time experience. These steps are practical and of particular interest to clinics, where waits are oftentimes inevitable.

## Background

Wait time, defined as time spent in the waiting and exam rooms waiting to see a provider, is a key quality metric in a number of national patient experience surveys (Table 1) [1–3]. However, the literature on wait time does not show a consistent correlation between long waits and worse overall patient care experiences [4–18]. In this study, we hypothesize that other factors (beyond actual wait time) may intervene to make a wait feel longer or shorter than it actually is, and explain inconsistent findings in the literature.

**Table 1:** Wait time items in patient experience surveys, by country

Country	Question
United States^a^	In the last 12 months, how often did you see this provider within 15 min of your appointment time?
	[Never / Sometimes / Usually / Always]
United Kingdom^b^	How long after your appointment time do you normally wait to be seen?
	[I don’t normally have appointments at a particular time / Less than 5 min / 5 to 15 min / More than 15 min / Can’t remember]
	How do you feel about how long you normally have to wait to be seen?
	[I don’t normally have to wait too long / I have to wait a bit too long / I have to wait far too long / No opinion or doesn’t apply]
Canada^c^	How long did you wait for your consultation to start?
	[Less than 5 min / 5 to 10 min / 11 to 20 min / 21 to 30 min / More than 30 min / There was no set time for my consultation]
	What type of difficulties did you experience?
	[Difficulty contacting a physician / A specialist was unavailable / Difficulty getting an appointment/ Do not have a personal or family physician / Waited too long to get an appointment / Waited too long in the waiting room / Service not available at the time required / Service not available in the area / Transportation problems / Cost issues / Language problems / Did not feel comfortable with the available doctor or nurse / Did not know where to go / Unable to leave the house because of a health problem / Other]

^a^Agency for Healthcare Research and Quality [http://www.ahrq.gov/cahps/surveys-guidance/cg/instructions/index.html]

^b^National Health Service [https://gp-patient.co.uk/Files/Questionnaire2018.pdf]

^c^Canadian Institute for Health Information [https://www.cihi.ca/sites/default/files/info_phc_patient_en.pdf]

Prior research on wait time in clinic has focused primarily on actual wait time. Some studies have found a weak correlation between long wait times and worse overall patient experiences [4–10]; others have found no correlation [11–19]. Only a handful have focused on perceived wait time, and most of these have taken place in the emergency and urgent care settings [18–22]. They indicate that perceived wait time can account for differences in patients’ evaluation of wait time and overall care experiences. For example, in Locke et al., multiple wait time variables that were statistically significant in bivariate analysis (i.e. actual wait time, child play activities in the waiting area, comfort of the waiting area), were not statistically significant after controlling for other variables (e.g. ratings of the doctor) [18]. In fact, “kept informed of delays” was the only wait time variable that remained significant. This study and others indicate that keeping patients informed of delays and providing positive experiences with the doctor can mitigate negative responses to a long wait. However, the data are quantitative and questions asked still do not represent the full spectrum of contextual factors (e.g. disease severity, health status, perceived value of the visit) that may influence how patients respond to different elements of the waiting experience.

Outpatient clinics are particularly well-suited to studying the wait time experience, where long waits do not result in adverse outcomes. In this environment, when waits are oftentimes inevitable, it is prudent for clinics to understand factors they may have control over that can improve the waiting experience.

In our study, we interview patients before and after their first visit with a provider at an HIV primary care clinic. Such interviews complement existing quantitative data, and permit a more in-depth examination of the wait time experience within a primary care context. Some of the pre-visit interviews occurred in real-time as patients waited, providing a unique window into how patients feel, think and respond to different contextual factors as the wait unfolds. Herein, we examine contextual factors and potential intervening variables that can shape the manner in which patients may respond to different waits. In addition, this study aims to identify actions providers and clinics can take to promote positive wait time experiences and mitigate negative ones.

## Methods

### Study population

Research staff contacted patients new to the HIV primary care clinics at the Michael E. DeBakey Veterans Affairs Medical Center (MEDVAMC) and Thomas Street Health Center (TSHC) in Houston, Texas. MEDVAMC is the VA’s 3rd largest HIV clinic, serving almost 1000 Veterans each year. TSHC is an urban, community-based HIV clinic, serving over 6000 patients yearly.

Study participants were recruited from August 2013 to July 2014 at MEDVAMC and August 2014 to November 2014 at TSHC. Eligible patients were: 1) older than 18 years of age, 2) diagnosed with HIV infection, and 3) had not yet completed their first visit with the HIV clinic. Exclusion criteria included those mentally unable to complete interviews or give informed consent, non-English speaking or incarcerated.

### Development and pre-testing of the interview guide

We developed an interview guide based on our prior work and a review of the literature. We pilot-tested the guide with five patients at MEDVAMC and 15 patients at TSC. We used the Think Aloud method to probe patients on their understanding of each question in our interview guide [23]. Participants received $20. Revisions to content and wording were made prior to the main study.

### Main study

This was part of a larger study to understand how new patients experience and evaluate their overall HIV care (NIH K23 MH100965) [24, 25]. We interviewed patients three times over their first year of HIV care (Table 2). The first interview occurred before the patient’s first visit with the HIV provider [T1]. The second occurred within 2 weeks after the first visit [T2]. The third occurred 6 to 12 months after the first visit [T3].

**Table 2:** Major topics and key questions, according to interview time point

Pre-visit	Now think about your first visit with the doctor at this HIV clinic	
	Hopes	Think about what things would be like if everything were perfect on the day of your first visit with the doctor at this HIV clinic. What do you hope will happen?
	Expectations	Now think about your first visit with the doctor at this HIV clinic. Walk me through everything you think will happen on the day of your first visit with the doctor.
		You step foot in the clinic. Now what?
		You’re sitting in the waiting room. Tell me about that. Now what?
		How long do you think you’ll have to wait?
		How long do you think the doctor will spend with you?
	Past experiences	You think you’ll wait [x] minutes. Has that been your experience elsewhere?
		Tell me about a doctor’s visit in the past. How long did you wait?
0–2 weeks post-visit	Last time we talked about your plans and expectations. Today, I would like to focus on how your visit actually went.	
	First impressions	How long did you wait?
		How did you feel about the wait?
		Tell me about the wait experience.
		How different or similar was this from what you thought?
		What did you do while you waited?
		How long did the doctor spend with you?
	Context	What did you like/not like about the clinic?
		Using any number from 0 to 10, where 0 is the worst clinic possible and 10 is the best clinic possible, what number would you use to rate this clinic?
		What are you thinking when you give a ___ rating?
		What would make you give a 10 rating?
	Actionable opportunities	What, if anything, could the clinic (or others) have done to make your experience at the clinic better?
		What, if anything, do you wish you had known before coming to the clinic for the first time?
		Is there anything the doctor could have done to make your experience better?
6–12 mos. post-visit	Last time we talked about how your first visit to the HIV clinic went. Today I’d like to talk about what’s gone on since that first visit. Tell me about your most recent visit with the HIV doctor.	
	Wait experience	How long did you wait?
		How did you feel about the wait?
		Tell me about the wait experience.
		What did you do while you waited?
		How long did the doctor spend with you?
	Context	What did you like/ not like about the most recent clinic visit?
		Using any number from 0 to 10, where 0 is the worst clinic possible and 10 is the best clinic possible, what number would you use to rate this clinic?
		What are you thinking when you give a ___ rating?
		What would make you give a 10 rating?
	Actionable opportunities	What, if anything, could the clinic (or others) have done to make your experience at the clinic better?
		Is there anything the doctor could have done to make your experience better?
		If you could change one thing about your HIV doctor, what would you change?

In the first interview, we asked patients about their ideals, hopes and expectations of wait times in the HIV clinic. We also asked about prior wait time experiences at other clinics. In the second interview, we asked patients about their first wait time experience at the clinic and how it aligned with their expectations. In the third interview, we asked patients about their most recent and overall wait time experiences. Findings emerged from patients’ stories praising positive wait time experiences and those voicing negative experiences. We probed patients for what they did not like about negative experiences and what they wish had happened instead.

Interviews took place in private rooms at MEDVAMC and TSHC or in community settings. Interviews were audio-recorded and professionally transcribed. Participants received $10 for completion of the first interview, $15 for the second interview and $25 for the third interview.

The Institutional Review Board at Baylor College of Medicine and the DeBakey VA Research and Development Committee approved this study. All participants gave written informed consent. All names in the text are pseudonyms to protect patient confidentiality.

### Data analysis

The core team consisted of two HIV primary care physicians and health services researchers with experience in qualitative research (B.N.D. and T.P.G.), a physician assistant student with experience volunteering at an HIV clinic in Cape Town, South Africa (H.C.), a Masters-level public health professional with formal training in qualitative methods (S.N.), and a business Professor with expertise in customer experience, satisfaction and retention and qualitative research (R.A.W.).

The principal investigator (B.N.D.) developed a list of codes based on a literature review, her prior work and notes taken during and shortly after each interview. The research team reviewed this list and developed definitions for each code’s use. ATLAS.ti software was used to code and evaluate interview data via conventional and directed content analysis [26]. The interview data were queried to identify quotes linked to the code for wait time. B.N.D. and H.C. reviewed the query reports and analyzed these data across time (all quotes for participants at T1, then T2 and finally T3) and across individual patient perspectives (all quotes in chronological order pertaining to each participant) [27]. B.N.D. and H.C. wrote memos regarding emerging themes related to wait time and noted memorable quotes. This information was frequently discussed as a team, and a consensus of the emergent themes was reached.

## Results

Fifty-six patients participated in this study (35 TSHC and 21 MEDVAMC patients). See Table 3. All completed the first interview, 48 (86%) completed the second interview and 34 (61%) completed the third interview. Interviews averaged 60 min each.

**Table 3:** Baseline characteristics of participants at Thomas Street Health Center and the Michael E. DeBakey VA Medical Center in Houston, Texas (*N* = 56)

Characteristics	
Age, years – mean (±SD)	56 (±13)
Gender – n (%)	
Male	30 (54%)
Female	26 (46%)
Race ethnicity – n (%)	
Non-Hispanic black	28 (50%)
Hispanic	14 (25%)
Non-Hispanic white	13 (23%)
Other	1 (2%)
Time from HIV diagnosis– (%)	
≤ 3 months	10 (18%)
3 months – 1 year	6 (10%)
1–5 years	13 (23%)
5–10 years	9 (16%)
> 10 years	18 (32%)
CD4 cell count < 200	12 (21%)
HIV RNA < 20 copies ^a^ – (%)	26 (47%)

### Factors affecting the perception of wait time

Our analyses of the patients’ wait time experiences revealed individual factors that may influence how patients perceive and respond to long and short waits.

#### Most patients expect to wait

Patients expected to wait to be seen by a provider, up to a certain point. Expectations varied widely, anywhere from a few minutes to an hour and with allowances for longer waits if an event beyond the control of the provider or clinic occurred. Patients based these expectations on their past experiences with the health care system and general norms of provider wait times. As patients wait, they compare their perception of the wait time length to these expectations. Notably, if the perceived wait time was shorter than expected, patients judged the length of their wait time as favorable. At the first interview, Sam (age 50s), talked about prior experiences waiting hours to see a provider. For the first HIV provider visit, the patient expected a similar “hurry up and wait” experience, and said, “[the wait] shouldn’t be no more than an hour.” The patient ended up waiting 35 min, less than expected, and judged the waiting time as favorable:

It didn’t take long at all …. It’s going better than I really expected it to go. You know, because I’m used to going to-you know, when I go to the clinic-other clinics I went to, it takes all day just to see [a doctor].

#### Patients rationalize that “things happen” and tend to be forgiving

Patients all hoped to have little if any wait. However, they rationalized that if the doctor were late, it was probably for something important or unavoidable. Lee (age 50s), said:

Doctor may [be in] traffic; he might be a little late. You know when uh- we don’t live in a perfect world.

Another patient, Jean (age 40s), said:

Joe may came in and had more issues than he coming in for in the first place, so they may have to spend more time with him.

Patients reported that understanding that “things happen” allowed them to wait with greater composure and patience.

#### Patients weigh the cost of waiting in their willingness to wait

Patients talked about the cost of waiting in terms of things they could be doing. Blake (age 40s) talked about wait time in terms of income lost:

That’s why I worry about how long it’s going to take because it’s like money, overtime money. Because I get 6 hours of overtime a day a week and that usually puts me in a survival range.

The more patients focused on what else they could be doing instead of waiting, the more aware they were of the passing of time. In contrast, patients who did not work or have other things they had to do did not mind waiting as much. Rowan (age 50s), said:

It’s just a process like everything else and they just time consuming but I don’t have nothing but time. Got more time than I got money.

Patients overall though, reported that the value of seeing a provider outweighed the value of any forgone activities. The above patient followed up and said “[I’m] not going to stress over it [the wait] too much because it’s more important to stay healthy.”

#### Patients who perceive greater value in a visit are willing to wait

New patients with life-altering illnesses feel vulnerable and anxious. Patients with high levels of anxiety or heightened concern reported that they were willing to wait to get their questions answered and reassurance that they will do okay. Avery (age 20s) who was recently diagnosed with HIV, reflected:

I felt nervous and anxious and I was scared … it’s a scary diagnosis … Because if I had to sit there until five o’clock that evening, I would’ve sat there ‘til five o’clock that evening just to be seen by the doctor … I needed clarity; I needed a peace of mind … And I’m blessed that I was able to be seen.

Even patients diagnosed with HIV for a long time can still feel vulnerable, and are willing to wait. Kendell (age 40s), who was diagnosed with HIV a decade ago, said:

I’m just so grateful that they help me. I’ll wait all day [to see a provider] if I need to … especially if it’s [for] a vital life-saving medication like I’m on. For the [VA] to be there for us … to give us the medication that we need that keeps us alive, it’s very emotional.

### Key opportunities for making wait times less stressful and more tolerable

Our analyses of the interviews revealed several steps providers and clinics can take to improve the wait time experience. Key steps include: 1) proactively informing patients of delays, 2) explicitly apologizing for delays, and 3) providing opportunities for diversion. Each step is detailed below.

#### Informing patients of wait delays reduces uncertainty and increases tolerance

Patients want to know how long they have to wait, especially with long waits. The uncertainty of not knowing can cause significant anxiety. Charlie (age 30s), talked during the first interview about picking up medicines at the clinic pharmacy:

“So I’m sitting here waiting … not knowing really which number - where I am in line because the numbers are random …. Like it will say - my number may be C851, and then they may call C734, and you’re thinking you’re coming later but then they say C724 …. I do think that they should definitely have the numbers in order if you’re gonna do that. It gives the person hope that they’re close.”

This patient kept hoping the pharmacy staff would call the patient next. However, the patient repeatedly felt let down when the staff called someone else. In this case, accurate queuing information could have let the patient more precisely estimate the wait time, and in turn, reduce uncertainty and distress.

Reese (age 20s), compared a negative wait time experience at a prior clinic, with a positive experience at the new clinic:

At the [prior] clinic, you’d go up there and ask them how long is the wait, and they’d be like just have a seat. We’ll get to you when we do. I mean, here it’s a lot more respectful. I’ll find out for you, or if you give me a minute, I’ll see how much longer the wait is, or I’ll see if I can bump you up if you’re in need of emergency.... Communication works really well around here.

At the prior clinic, the patient felt dismissed when the front desk staff said, “Just have a seat.” Patients want clinic staff to take their inquiries seriously and investigate. Another patient Casey (age 40s), said during the third interview:

You don’t have to wait so long and then if it’s a delay they’ll come out and tell you it’s a delay. So that’s a good thing. They’re letting you know what the delay is like, she delayed three patients or how many patients and that you know she fell behind because the new patients coming in.

This patient appreciated receiving continuous updates on the place in line. Even though the doctor had three more patients to see, the patient did not seem upset. In fact, the patient accepted the situation and reported a positive overall wait time experience.

#### Apologizing for delays can mitigate negative emotions arising from a long wait

With excessively long waits that exceed even low expectations, patients can experience a variety of negative emotions, such as anxiety, irritation, anger and frustration. In these instances, explicit and sincere apologies can go a long way in relieving negative emotions. Our interview with Jordan (age 50s), took place during an excessively long wait, and uniquely captures the patient’s emotions in real-time, as the events unfolded. This patient waited almost 2 hours before learning that the scheduled provider no longer worked in the clinic. The patient finally asked a nurse, who responded ‘Just go sit down, they’ll call.’ The nurse’s dismissive attitude angered the patient. The patient persisted:

I said, “Who is my doctor?” “They’ll call you.” …. I don’t know how she made charge nurse, but I don’t like her.... You don’t tell me. I’m a patient; I asked you a question then answer it. Don’t tell me to go sit down and- and- I was offended….

The patient also felt incredibly angry at the provider.

If I had fire in my eyes, I’d’ve burnt that doctor …. My time is valuable. Don’t waste my time.

However, the patient’s anger subsided when a resident doctor came out and apologized:

That [other] doctor c[a]me out and apologize[d] … you know how they say um, you dropped the ball and someone had your back.

This provider stepped in, saw the patient and completed the initial visit. In fact, the patient ended up rating the provider experience a 10 out of a maximum of 10:

He covered everything from A to Z and I thought that was great. For- as you know for having to fill in behind another doctor and- and me being the patient and pissed off; I think he did pretty good. He kept apologizing, “Apologies; I’m so sorry, I really am.”

The provider’s apology and acknowledgement of the patient’s anger mitigated a negative situation and calmed the patient. At the third interview, nine and a half months later, the patient recalled the incident:

[The fill-in doctor] said, “Oh I can tell you’re not happy.” “No I’m not.” I said, “It’s not your fault, I understand it but guess what. You’re the doctor they put me, so you’re the doctor that’s going to hear it.” But ever since then it’s been fine.

#### Create opportunities for patients to use wait time constructively

Many patients expressed a desire to spend their wait time productively or enjoyably. Wyatt (age 40s), said:

Wait time is a big thing because it’s non-productive time and non-useful time in my eyes because I’m sitting there twiddling my thumbs waiting. If they filled my wait time with something to do maybe- maybe it would not be so wasted.

Patients talked about coming prepared, with a book or device, such as a smart phone, tablet, or laptop. Others talked about reading pamphlets, magazines and “new posters on the wall,” looking at artwork, or socializing with other patients in the waiting room. Patients also talked about wanting to have the ability to leave the waiting room and come back. Emerson (age 20s), did not know there was a change in appointment time and unknowingly checked-in several hours early. However, the front desk staff did not inform the patient of the misstep:

[She] didn’t notify me; didn’t question the fact of why I was checking into an appointment three and a half hours early. Just let me sit. Um just you know make me aware if they have any changes that- to my schedule. I don’t have a problem with walking around or going somewhere or you know for three and half hours.

This patient’s regret was not necessarily the long wait, but rather, that the patient could have used that time to do something beneficial. Other patients talked about not minding long waits if they could safely leave and “get some coffee” or “breakfast” “to pass idle time.” However, unless told when to come back, patients worry about losing their place in line if they leave the waiting room, even if only to use the restroom. Ashley (age 40s), said:

I was worried about OK if they coming out looking for me then I’m gonna miss my spot.

## Discussion

This qualitative study provides insight into the psychology of the wait time experience – that is, how patients feel and think about time spent in the waiting and exam rooms waiting to see a provider. Our study showed that patients’ “willingness to wait” can vary depending on a variety of factors beyond actual wait time. These include contextual factors, such as the perceived value of the visit and the costs of a long wait, and clinic and provider factors. These latter set of influences is of particular interest, since clinics and providers can manage these to improve wait time experiences for patients. Specifically, clinics and providers can: 1) proactively inform patients of delays, 2) explicitly apologize for delays, and 3) provide opportunities for diversion.

**Fig. 1:**
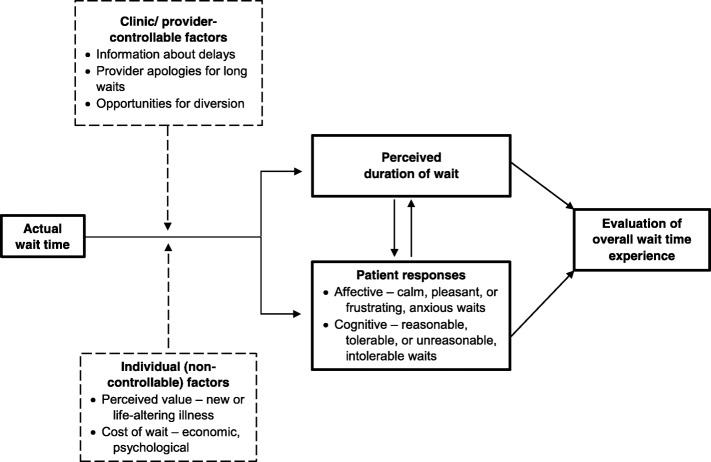
Key variables in the patient’s overall wait time experience. The variables in the dotted line boxes are proposed moderators of the relationships between: a) Actual Wait Time and Perceived Duration of Wait and b) Actual Wait Time and Patient Responses, i.e., they affect the direction or strength of each pair of relationships

Figure 1 illustrates a model of the wait time experience, developed from our analysis of the qualitative interviews. It highlights specific steps that clinics and providers can take to improve patients’ wait time experience, while the wait is taking place in real time and even after it has passed. Patients noted the importance of these steps in curtailing frustrations that may result from a long wait and in mitigating negative wait time experiences.

Our study showed that many patients tolerate some degree of wait time. However, when the wait time sufficiently exceeds patients’ expectations or norms, and is judged excessive, patients want their provider to acknowledge this delay. Such an acknowledgement can mitigate a negative wait experience. Acknowledging delays serves two purposes [28]. First, it lets the patient know that the provider recognizes delays as an unwanted event that neither party desires and that frustration and anger are understandable reactions to it. Second, it shows that the provider respects the patient’s time, cares about what the patient thinks and does not want the patient to wait unnecessarily.

One of the most frustrating aspects of waiting is the uncertainty of the wait length. Uncertainty can cause angst and make waits seem even longer. Information on delays can reduce uncertainty and make the patient perceive the wait as something manageable, and in turn, more tolerable [10, 18, 20, 21]. Patients also feel a greater sense of control because they can cognitively reappraise the situation and adjust their expectations, such that the wait then feels more predictable [29, 30]. Knowing what to tell patients in waits of different lengths may also reduce stress and create greater tolerance. In a study of consumer reactions to different wait lengths, consumers were less irritated and more accepting of a long wait (i.e. in waits longer than 15 min) when given queuing information (e.g. their position in line), as opposed to an estimated wait time [30, 31]. This approach may apply to clinics, where a physical line does not exist and it is difficult to accurately estimate the actual wait time.

Applications that reduce the uncertainty of wait times have been shown to reduce perceived wait time and stress in a variety of service sectors. Several sectors have adopted mobile applications and text messaging services: restaurants (e.g. No Wait, Waitlist Me), government agencies like the Department of Motor Vehicles (e.g. Dash Pass) and the Department of Public Safety (e.g. QLess), and amusement parks (e.g. My Disney Experience) [32–35]. These platforms update patrons on their wait times, letting them readjust their wait time expectations and engage in productive activities during their waits (e.g. they can leave and come back or they can do other things). Similar tools appear to have notable potential in health care settings, although they have yet to become widely used [36, 37].

Strategies to fill wait time with active activities serve to engage the patient and divert attention from the passage of time [38]. Data suggest that related fillers may improve the overall wait time experience more than unrelated fillers [39, 40]. In health care, this can entail reorganizing the work flow, such that patients complete necessary health related tasks while waiting to see the provider. For example, nurses can administer scheduled vaccines, or patients can be sent to get missing labs or other diagnostic studies as appropriate. Using wait times constructively may decrease the total time in clinic and have the added benefit of decreasing perceived wasted time and boredom and making the wait experience more pleasant.

Aside from clinic- and provider-controlled factors, contextual factors also play a role in the wait time experience for patients. The perceived value of the visit can vary for patients with differing characteristics. For example, patients newly diagnosed with a life-altering illness, such as HIV infection or cancer, may approach a visit with greater anxiety and vulnerability and thus, be more willing to wait [41]. Apart from the perceived value of a visit, the economic or psychological cost of a long wait can have a substantial impact on a patient’s wait time experience [30, 42]. For example, patients whose jobs pay on an hourly basis can face a significant economic cost in waiting. Similarly, a parent with restless children endures a psychological cost in waiting. Costs such as these and others can evoke negative emotions and make the wait time seem longer. Although some studies exist, more empirical research is needed to evaluate the mechanisms through which cost-benefit appraisals and other contextual factors may impact the wait time experience [43].

A major strength of this work is its longitudinal design. Our chronicle of Jordan’s story, in particular, comparing quotes at times T1, T2, and T3, uniquely chronicled the patient’s emotions as they unfolded in real-time. This methodology is novel and unlike previous studies, which frequently asked about the wait time experience once the actual wait was over.

This study has a few limitations. The study took place in the context of primary care, and results may not translate to non-clinic settings. The study population included predominantly older men with public insurance, which may not generalize to those who are younger, female or with private insurance. Furthermore, the results of our research, which focused on patients with HIV infection, may not apply to less vulnerable disease populations. Nevertheless, the findings still add insight into the wait time experience of patients with chronic medical conditions. Lastly, although 86% of patients had a second interview, only 61% of patients completed the third interview. However, even with this longitudinal drop-off, we still had 34 participants at T3. Studies indicate that data saturation can occur with as few as 12 participants, especially when populations are similar [44]. In our study, all patients were new to the provider, and we had no issues reaching thematic saturation.

## Conclusion

This study identified several modifiable factors affecting patients’ perceptions of their wait time, all of which were salient and consequential to the favorability of their overall waiting experience. Perhaps equally, if not more important than efforts to cut down the actual wait time, are efforts to change the perception of those wait times. The wait time experience is an actionable target that is an attainable and feasible focus for practice management and process improvement.

## Data Availability

This is a qualitative study with full length interviews. Requests to view de-identified interview data will be considered on a case-by-case basis, following written request to the Principal Investigator.

## Abbreviations

MEDVAMC: Michael E. DeBakey Veterans Affairs Medical Center
TSHC: Thomas Street Health Center
